# A meta-analysis of the effect of Sjögren′s syndrome on adverse pregnancy outcomes

**DOI:** 10.1016/j.clinsp.2022.100140

**Published:** 2022-11-17

**Authors:** Baoqing Geng, Keyue Zhang, Xianqian Huang, Yong Chen

**Affiliations:** Department of Rheumatology and Immunology, Ningbo Huamei hospital, University of Chinese Academy of Sciences, Ningbo, Zhejiang, China

**Keywords:** Sjögren's syndrome, Pregnancy outcome, Meta-analysis

## Abstract

**Objective:**

This study aimed to assess the correlation between Sjögren′s Syndrome (SS) and adverse pregnancy outcomes, with the aim of providing a basis for preconception and pregnancy interventions in women with SS.

**Methods:**

A search of electronic databases in English and Chinese databases from January 2005 to December 2021, was conducted to collect the literature of case-control studies or cohort studies on the association between SS and pregnancy outcome studies. Literature inclusion and data extraction were performed according to established criteria, and the Newcastle-Ottawa scale was used to evaluate the quality of the literature. Stata 15 software was used for meta-analysis.

**Results:**

A total of nine papers were included in this study. Meta-analysis results showed that SS was associated with spontaneous abortion (RR = 8.85, 95% CI 3.10‒25.26), preterm birth (RR = 2.27, 95% CI 1.46‒3.52), low birth mass (RR = 1.99, 95% CI 1.34‒2.97), and birth defects (RR = 4.28, 95% CI 3.08‒5.96).

**Conclusion:**

SS can increase the incidence of adverse pregnancy outcomes.

## Highlights

• Dry syndrome can increase the risk of spontaneous abortion and preterm delivery.
• Dry syndrome can increase the risk of low birth weight and birth defects.
• Clinics should provide comprehensive prenatal counseling and testing for the patients.

## Introduction

Sjögren′Syndrome (SS) is a systemic immune disease, mainly characterized by the impairment or absence of secretory gland function leading to pathological damage in the oral cavity and eyes, and also involving other tissues and organs of the body. Antibody testing revealed positive anti-Ro/SSA and anti-La/SSB antibodies.^1^ When SS occurs alone, it is primary Sjögren′s Syndrome (pSS), and when it coexists with other connective tissue diseases such as Rheumatoid Arthritis (RA), it is secondary Sjögren′s syndrome (SSS).^2^ The prevalence of SS ranges from 0.29% to 0.77% and is significantly higher in women than in men (gender ratio: 1.9:20), making it a life-threatening event for women.^3^

SS is an autoimmune disease with a good prognosis, and most of them can be remitted after standardized treatment, but patients with combined visceral damage can experience relapse after discontinuation of the drug. In early clinical practice, most SS patients were diagnosed at the age of 40‒50 years, so its effect on pregnancy was not taken seriously. However, in recent years, women's reproductive age has been higher, coupled with the enhanced public awareness of early diagnosis and early treatment, more than 25% of SS patients are diagnosed before the age of 35,^4^ so its impact on pregnancy has gradually been paid attention to. Recent studies have shown that SS may be associated with adverse pregnancy outcomes, and those antinuclear antibodies, anti-SSA, and anti-SSB in women during pregnancy may affect fetal development and increase the risk of adverse pregnancy outcomes such as miscarriage, congenital heart block, and preterm delivery.^5^

In this study, the authors intend to investigate the effect of SS on pregnancy outcomes through a literature review and meta-analysis in order to provide a basis for the management of women with SS during pregnancy.

## Materials and methods

### Literature search strategy

The databases including CNKI, Wanfang Database, Cqvip Database, PubMed, Web of Science, Embase and Cochrane Library were searched. The search terms were as follows: (1) SS; (2) Pregnancy, pregnancy outcome; (3) Spontaneous abortion, preterm birth, low birth weight, congenital malformation, birth defect, congenital heart disease, congenital heart block; the combination of (1)+(2) or (1)+(3) was searched. The NoteExpress library was used for the removal of duplicate articles. This study is a literature review and has no ethical implications. The review was conducted according to PRISMA 2020 statement.^6^

#### Inclusion criteria

(1) A case-control study or a cohort study; (2) The publication time of the literature: January 2005 to March 2022; (3) The patients in the case group were diagnosed with pSS, and the control group consisted of healthy pregnant women; (4) The literature was in Chinese or English; (5) At least one of the following outcomes was described: (a) Spontaneous abortion: miscarriage within 28-weeks of gestation without any interventions; (b) Birth defect: morphological, structural and functional abnormalities, including congenital heart disease, Down's syndrome, harelip, thalassemia, malformations of the digestive system, malformations of the genitourinary system polydactyly, etc.; (c) Low birth weight: < 2500g; (d) Premature birth: gestational age < 37-weeks.

#### Exclusion criteria

(1) Duplicate reports for the same study population. In this case, the literature with the largest sample size was included; (2) Study subjects with other autoimmune diseases, such as systemic lupus erythematosus; (3) Studies with design flaws; (4) Incomplete data on outcome indicators; (5) Incorrect statistical methods; (6) Literature review studies, conference reports, animal experiments, clinical interventional studies.

### Literature data extraction

Two investigators independently conducted literature screening and extracted data from the included literature and negotiated to resolve disagreements. A data collection form was developed, and the extracted information included authors, year of publication, study duration, sample size, age of study subjects, and outcome indicators.

### Risk of bias assessment

The Newcastle-Ottawa Scale (NOS),^7^ which includes three dimensions of study subject selection, comparability between groups, and exposure factor measurement, was used, with a maximum score of 9. A score of ≥ 7 was considered high-quality literature.

### Statistical analysis

Stata15 software was used for data analysis. Relative Risk (RR) was used as an effect statistic for each outcome indicator (spontaneous abortion, preterm birth, birth defects, and low birth weight). A Chi-Square test was used to analyze the heterogeneity of the literature, combined with I^2^ values. Outcome indicators with p > 0.1 and I^2^ < 50% were used for meta-analysis using a fixed-effects model; outcome indicators with p < 0.1 and I_2_ > 50% were used for meta-analysis using a random-effects model. Publication bias was analyzed using Begg's test with Egger's test. Statistical significance was considered at p < 0.05.

## Results

### Basic characteristics of the included literature

**Figure 1:**
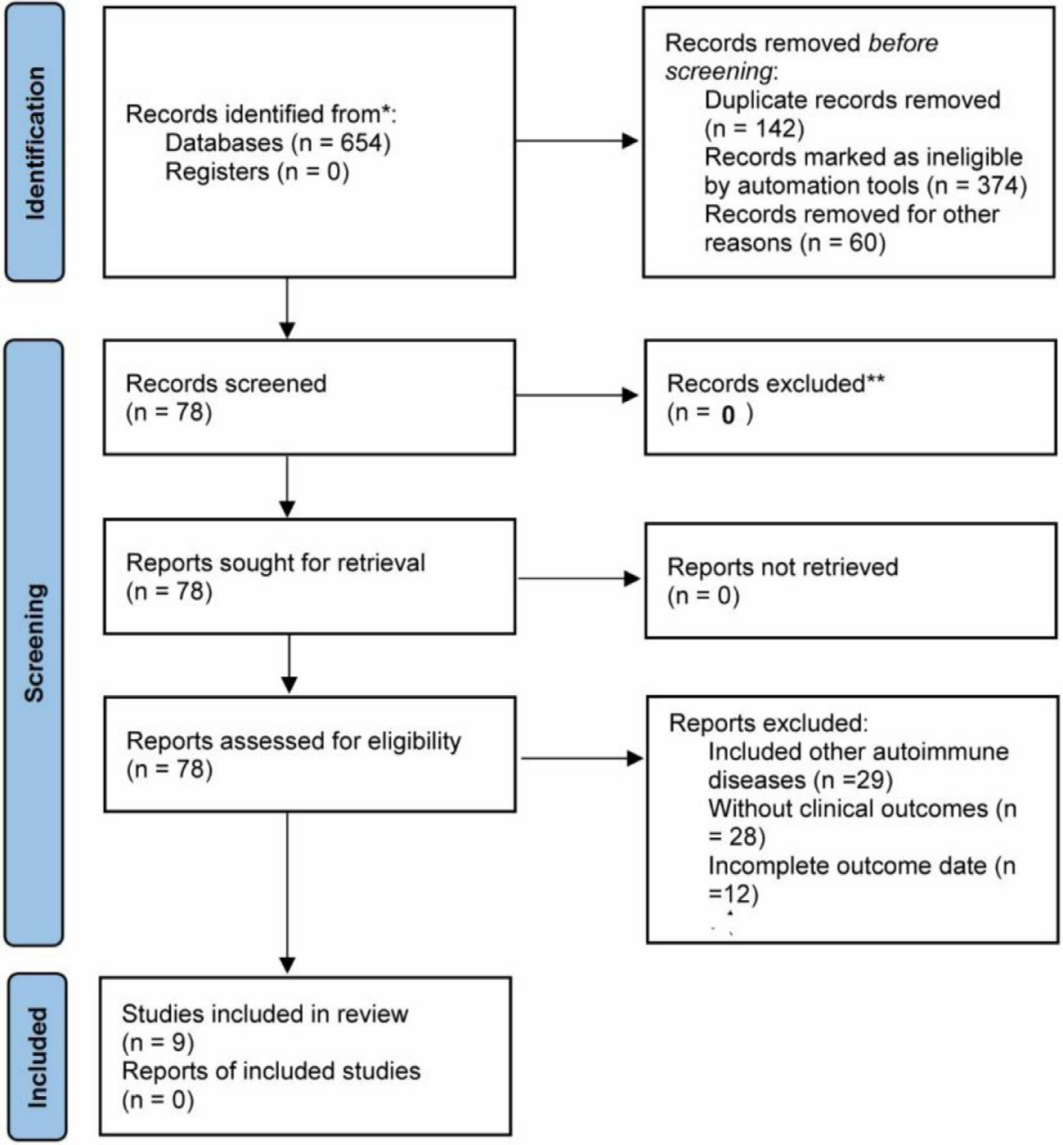
The PRISMA chart for presenting the flow of the included papers.

The study cumulatively included nine eligible papers, two in Chinese and seven in English. Two were prospective studies^8^^,^^9^ and seven were retrospective studies.^10^, ^11^, ^12^, ^13^, ^14^, ^15^, ^16^ NOS scores: 1 with a score of 5, 3 with a score of 6, and 5 with a score of 7; 5 pieces of literature were of high quality (≥ 7). The PRISMA flowchart is shown in Figure 1, and the basic information of the included literature is shown in Table 1.

**Table 1:** Basic characteristics of the included literature and NOS results.

Author	Year of publication	Study type	Age (years)		Sample size		Pregnancy outcome	NOS
			Case group	Control group	Case group	Control group		
C Ballester^11^	2017	Retrospective	30.8±6.2	30.8±6.2	54	216	Spontaneous abortion, preterm birth, low birth weight	7
Li L^9^	2019	Prospective	30.4±5.3	31.6±5.2	48	96	Spontaneous abortion, preterm birth	7
S De Carolis^12^	2014	Retrospective	34.8	30.2	34	136	Spontaneous abortion, preterm birth, low birth weight	6
R Priori^13^	2014	Retrospective	Not described	Not described	12	96	Premature birth	5
B Elliott^10^	2019	Retrospective	Not described	Not described	1 947	14 511 640	Premature birth, congenital malformations	6
Yang, Jingjing^8^	2021	Prospective	31.89±3.55	30.81±3.73	56	260	Spontaneous abortion, premature birth, and	7
HJ Haga^14^	2005	Retrospective	26	26	36	93	Spontaneous abortion, preterm birth, low birth weight, congenital malformations	7
ZE Hussein^15^	2011	Retrospective	33.6±4.2	29.8±5.5	16	80	Spontaneous abortion, preterm birth, low birth mass, congenital malformations	7
JF Xu^16^	2019	Retrospective	32.0±4.3	30.3±4.1	64	320	Spontaneous abortion, preterm birth, low birth mass, congenital malformation	6

### Spontaneous abortion

**Figure 2:**
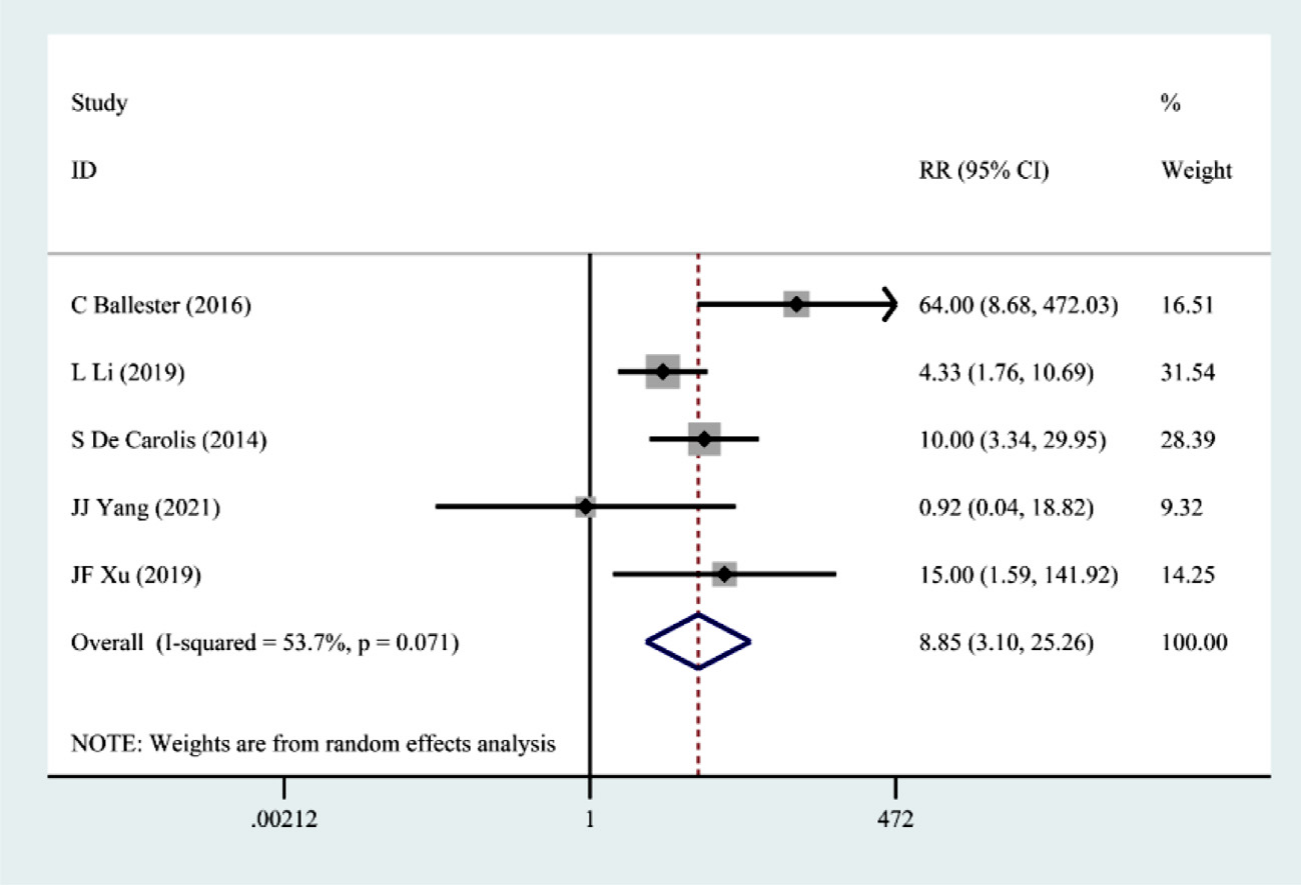
Correlation between pSS and spontaneous abortion.

Five papers^8^^,^^9^^,^^11^^,^^12^^,^^16^ described the occurrence of spontaneous abortion in pregnant women with pSS, and the results of the random effects model showed a significantly increased risk of spontaneous abortion in pregnant women with pSS (RR = 8.85, 95% CI 3.10 ∼ 25.26) (Fig. 2).

### Preterm birth

**Figure 3:**
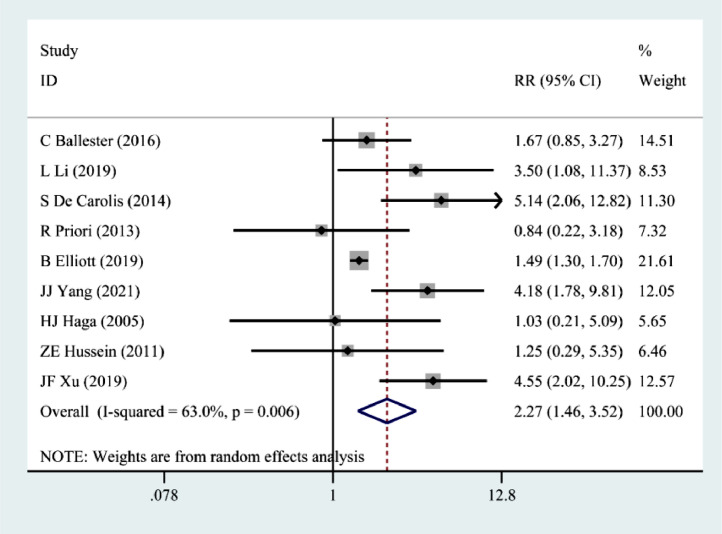
Correlation between pSS and preterm birth.

Nine papers^8^, ^9^, ^10^, ^11^, ^12^, ^13^, ^14^, ^15^, ^16^ described the association of pSS with preterm birth. The random effects model showed that pSS significantly increased the risk of preterm birth (RR = 2.27, 95% CI 1.46‒3.52) (Fig. 3).

### Low birth weight

**Figure 4:**
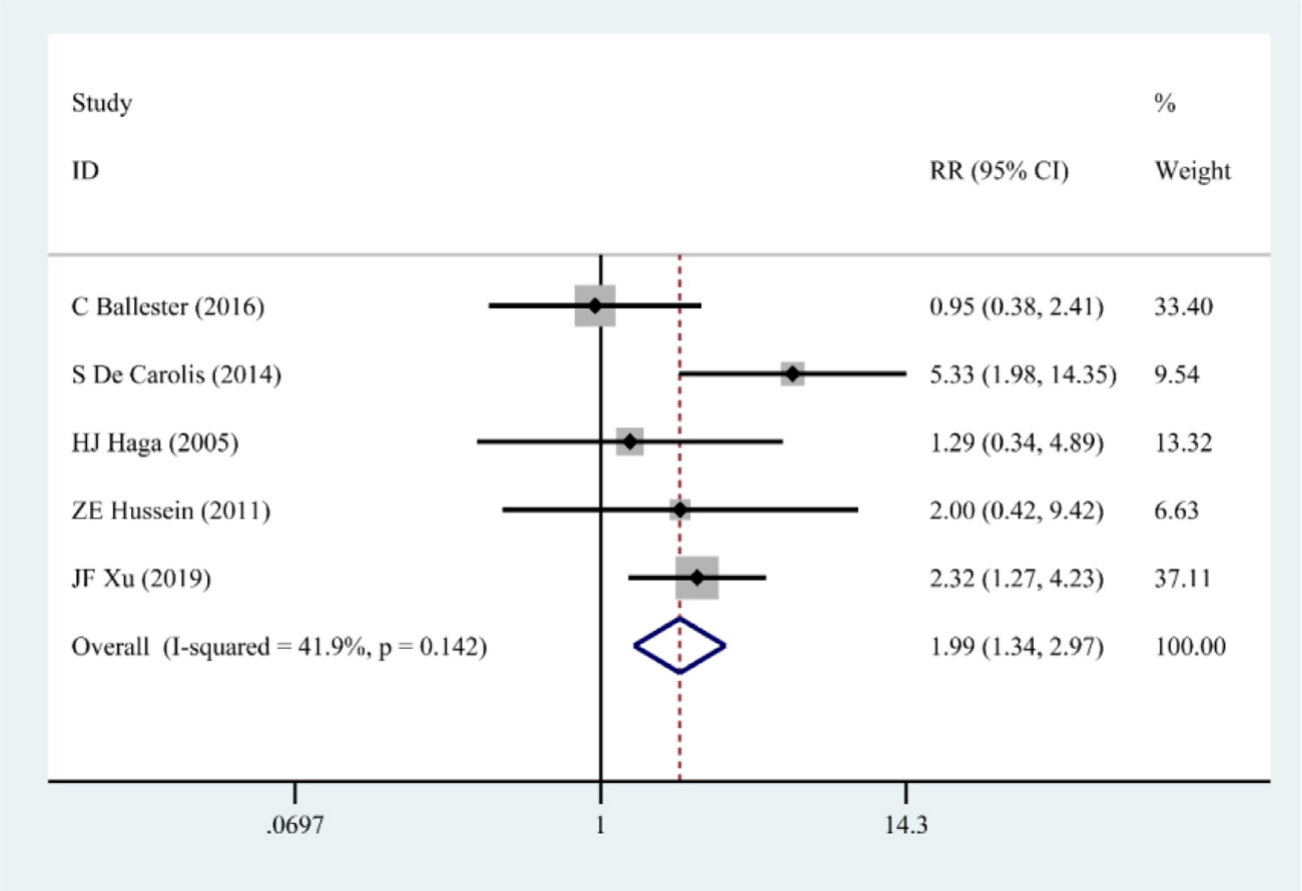
Correlation between pSS and low birth weight.

Five papers^11^^,^^12^^,^^14^, ^15^, ^16^ describe the correlation between pSS and low birth mass. The results of the fixed effects model showed that pSS increased the risk of low birth mass (RR = 1.99, 95% CI 1.34‒2.97) (Fig. 4).

### Birth defects

**Figure 5:**
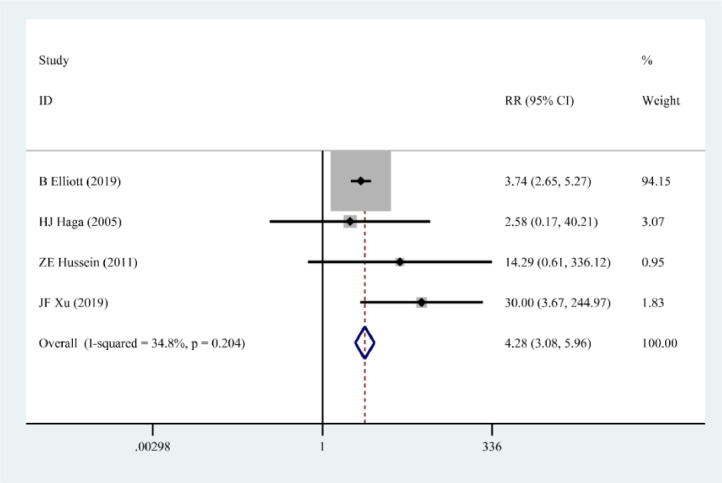
Correlation between pSS and birth defects.

Four papers^10^^,^^14^, ^15^, ^16^ describe the effect of pSS on the occurrence of birth defects. The results of the fixed-effects model showed that pSS significantly increased the risk of birth defects (RR = 4.28, 95% CI 3.08‒5.96) (Fig. 5).

### Sensitivity analysis

In the meta-analysis of the correlation between pSS and spontaneous abortion, preterm birth, low birth mass, and congenital malformation, the literature with the largest RR value and the smallest RR value were excluded and then meta-analysis was performed to compare the RR values with 95% CI before and after the exclusion, and the results are shown in Table 2. The results of sensitivity analysis showed that the correlation between pSS and spontaneous abortion, preterm birth, low birth weight, and congenital malformation was still statistically significant after the exclusion of literature, and the changes of RR values were relatively small, suggesting that the results of the original meta-analysis were reliable.

**Table 2:** Results of sensitivity analysis.

Adverse pregnancy outcome	Before sensitivity analysis RR (95% CI)	Exclusion of literature with the largest RR		Exclusion of literature with the smallest RR	
		Exclusion of literature	RR after exclusion (95% CI)	Exclusion of literature	Post-exclusion RR (95% CI)
Spontaneous abortion	8.85 (3.10‒25.26)	C Ballester	5.73(3.09∼10.65)	Yang Jingjing	10.99(3.79, 31.82)
Premature birth	2.27 (1.46, 3.52)	JF Xu	2.04 (1.33, 3.23)	R Priori	2.46(1.54, 3.92)
Low birth weight	1.99 (1.34, 2.97)	S De Carolis	1.74 (1.11, 2.73)	C Ballester	2.52 (1.61, 3.94)
Birth defects	4.28 (3.08, 5.96)	JF Xu	3.77 (2.69, 5.29)	HJ Haga	8.25 (1.81, 37.61)

### Analysis of publication bias

The publication bias was quantified using Begg's test and Egger's test, and the results of Egger's test showed that there was a publication bias in the meta-analysis of the correlation between pSS and spontaneous abortion (p = 0.016), while there was no publication bias for the rest of the outcome indicators (p > 0.05). The specific data are shown in Table 3.

**Table 3:** Results of publication bias.

Outcome indicators	Begg's test		Egger's test	
	*Z*	p	*t*	p
Spontaneous abortion	1.594	0.094	3.168	0.016
Premature birth	0.453	0.657	0.951	0.371
Low birth weight	0.395	0.693	1.727	0.136
Birth defects	0.737	0.451	1.238	0.263

## Discussion

SS can lead to the involvement of many exocrine glands and tissues, and the placenta is also one of the target organs during pregnancy, and placental dysfunction can occur after injury, and maternal IgG such as anti-SSA, anti-SSB, and anti-nuclear antibodies can also enter the fetus through the placental barrier and affect its intrauterine development, leading to a variety of adverse pregnancy outcomes such as miscarriage, congenital malformations, stillbirth, and preterm delivery.^17^ SS can also increase the risk of multiple pregnancy comorbidities, such as preeclampsia and premature rupture of membranes, and increase the incidence of complications such as postpartum deep vein thrombosis, thereby affecting maternal health and pregnancy outcomes.^10^ Pregnancy itself can also have an impact on SS progression, with approximately 30% of SS patients experiencing disease exacerbation during pregnancy, causing significant increases in anti-SSA and anti-SSB levels, and decreases after the termination of pregnancy.^18^ It has also been found that a small percentage of women with SS can also develop anti-erythrocyte antibodies during pregnancy, along with the aggregation of cytokines such as IL-4, leading to comorbidities such as hemolytic anemia and lymphohistiocytic hyperplasia.^19^ All of these phenomena can negatively affect pregnancy and increase the risk of adverse pregnancy outcomes.

Some studies have evaluated the association of pSS with adverse pregnancy outcomes, but its risk is difficult to assess relative to the general population, mainly because of the high number of confounding factors such as age, body mass index, and comorbidities, and the difficulty of dealing with confounding factors. C Ballester et al.^11^ balanced common confounding factors and found that pSS was an independent risk factor for spontaneous abortion. It is now believed that women with pSS have significantly prolonged menstrual cycles, decreased pregnancy success, and can develop immune infertility.^20^ pSS is a common cause of immune infertility, where antinuclear antibodies, anti-SSA, and anti-SSB bind to the antigen and attack the embryo, leading to difficulty in implantation or miscarriage.^21^ A systematic review with the meta-analysis by Upala et al.^22^ showed that there was an increased risk of neonatal death and fetal loss in pregnant patients with pSS, but there was no statistical difference in the incidence of premature birth, or stillbirth between pSS and normal pregnant women. The number of papers included in the study by Upala et al.^22^ was fewer than that in this study, and half of them were published before 2000, and the incidence of birth defects and low birth mass was not reported. The present study enrolled the papers in recent years and analyzed the common adverse pregnancy outcomes related pSS. The correlation between pSS and spontaneous abortion was reported in five papers in the present study, and the results of the meta-analysis showed that the risk of spontaneous abortion in pSS pregnant women was 8.85 times higher than that of healthy pregnant women, and the risk of spontaneous abortion in pSS pregnant women was significantly increased. The above results suggest that clinical interventions should be actively carried out in women with SS during the preparation period and early pregnancy, but it is uncertain whether SS-related clinical interventions can improve pregnancy success and reduce the spontaneous abortion rate, and further clinical studies are needed to provide evidence.

SS can also have multiple negative effects on neonatal outcomes. Compared to healthy pregnant women, SS women have shorter gestation times, a higher incidence of preterm delivery, a lower percentile birth weight, and a higher proportion of Small for Gestational Age (SGA) infants. A cohort study by Chen JS et al.^23^ showed an increased incidence of acute adverse events such as hypertensive disorders and cerebral hemorrhage during pregnancy, a significantly higher incidence of preterm delivery and low birth weight, and a corresponding increase in the cesarean delivery rate in SS and SLE pregnant women. On one hand, SS can directly damage the placenta and affect fetal development and health; on the other hand, SS can increase the risk of complications and adverse events during pregnancy, all of which can lead to adverse pregnancy outcomes such as preterm delivery, low birth weight, neonatal asphyxia, and hypoxic-ischemic encephalopathy. The present study showed that pSS pregnancies experience an increased incidence of preterm delivery and low birth weight, and this correlation remained significant in sensitivity analysis, which further confirms the effect of SS on adverse pregnancy outcomes.

It was found that SS can also increase the risk of birth defects such as congenital heart disease.^8^ Circulating antibodies in pregnant women with SS can cause the development of congenital heart block in the fetus, requiring pacemaker implantation in the newborn in severe cases.^24^ Therefore, the functional and structural characteristics of the fetal/neonatal heart should be highly monitored during the perinatal period and appropriate interventions should be given in a timely manner. Antibodies such as antinuclear, anti-SSA, and anti-SSB are the main antibodies mediating tissue damage in SS patients and are important factors predisposing to pregnancy complications. These antibodies can cross the placenta at 12 weeks of gestation and induce myocarditis and arrhythmias after acting on fetal myocardial tissue.^25^ In a study by Doti PI et al.^26^, the risk of congenital conduction block in the fetus of a pregnant woman with SS was higher than in pregnant women with other autoimmune diseases. Studies have shown that human fetal heart perfusion with anti-SSA can lead to transient heart block, mainly probably because anti-SSA can cross-react with T- and L-type calcium channels and the aforementioned reactions can also cause structural damage to the heart.^27^^,^^28^ It has also been shown that fetal atrial wall and annular echogenicity enhancement on ultrasound during pregnancy is associated with SS.^29^ The current study showed that pSS increased the risk of birth defects, suggesting that for pregnant women with SS, obstetric clinics should enhance prenatal consultations and early detection of congenital malformations to reduce birth defects.

In conclusion, SS can increase the risk of spontaneous abortion, preterm delivery, low birth weight, and birth defects, and the clinic should provide scientific and comprehensive preconception counseling and prenatal testing for female patients with SS, actively treat SS and gestational comorbidities, detect birth defects at an early stage, and choose the appropriate timing and mode of pregnancy termination to improve the prognosis of pregnant women and newborns. However, it is important to note that the existing studies on the association between SS and adverse pregnancy outcomes are relatively few, and most of the existing studies have failed to adequately manage confounding factors. On the other hand, this meta-analysis did not include articles written in other languages except for Chinese and English, which may lead to selection bias. Researchers still need to further analyze the association between SS and adverse pregnancy outcomes through high-quality cohort studies to provide a basis for the development of clinical intervention strategies.

## Authors’ contributions

Baoqing Geng, Keyue Zhang and Yong Chen designed and performed the experiments. Xianqian Huang and Yong Chen performed the experiments and analyzed the data. Baoqing Geng and Yong Chen wrote and revised the manuscript.

## Funding

No funding was received.

## Conflicts of interest

The authors declare no conflicts of interest.
